# Ten years of the *The Dietary Guidelines for the Brazilian Population*: history, science and policy

**DOI:** 10.1590/S2237-96222025v34e20240267.en

**Published:** 2025-03-31

**Authors:** Patricia Constante Jaime, Murilo Bomfim Lobo Braga

**Affiliations:** 1 Universidade de São Paulo, Núcleo de Pesquisas Epidemiológicas em Nutrição em Saúde, São Paulo, SP, Brazil

**Keywords:** Food Guides, Nutrition Policy, Health Promotion, Brazil, Narrative Review, Guías Alimentarias, Política Nutricional, Promoción de la Salud, Brasil, Revisión Narrativa

## Abstract

**Objective:**

This article organizes the origin and historical context of the second edition of the *Dietary Guidelines for the Brazilian Population*ten years after its publication, analyzing its contributions to the science of Nutrition and public food and nutrition policies in Brazil and around the world.

**Methods:**

This is a narrative review prepared based on consultation of scientific articles, gray literature and journalistic publications, among others.

**Results:**

The Guide resulted from scientific evidence and subsequently became an object of study. Its being based on the Nova Food Classification brought a new paradigm to nutritional science. Its publication was the basis of a series of public policies that encourage consumption of fresh or minimally processed foods and mitigate consumption of ultra-processed options.

**Conclusion:**

The Guide was able to induce and guide health actions and other public policies to promote adequate and healthy eating. It also boosted scientific research, having national and global influence.

## Resumen

**Objetivo:**

Este artículo organiza el origen y contexto histórico de la segunda edición de la *Guía Alimentaria para la Población Brasileña* diez años después de su publicación, analizando sus contribuciones a la ciencia de la Nutrición y a las políticas públicas de alimentación y nutrición en Brasil y en el mundo.

**Métodos:**

Se trata de una revisión narrativa elaborada a partir de la consulta de artículos científicos, literatura gris y publicaciones periodísticas, entre otros.

**Resultados:**

La Guía surgió de la evidencia científica y posteriormente se convirtió en objeto de estudio. Su base en la Clasificación Nova de Alimentos trajo un nuevo paradigma a la ciencia nutricional. Su publicación fue la base de una serie de políticas públicas que incentivan el consumo de alimentos frescos o mínimamente procesados ​​y mitigan el consumo de opciones ultraprocesadas.

**Conclusión:**

La Guía logró inducir y orientar acciones de salud y otras políticas públicas para promover una alimentación adecuada y saludable. También impulsó la investigación científica, teniendo influencia nacional y global. Palabras clave: Guías Alimentarias; Política Nutricional; Promoción de la Salud; Brasil; Revisión Narrativa

## Introduction

Over the last 15 years, in Brazil and other countries (especially in Latin America), we have seen a change in the food and nutrition paradigm. For decades, nutrition teaching, research and policy were based on a line of thinking centered on nutrients: foods were classified by their macro- and micronutrient content, and nutritional guidance aimed to ensure intake of minimal amounts of carbohydrates, proteins and fats, which made sense for most of the 20th century (1-4).

The epidemiological transition recorded in the last 15 years has involved overcoming infectious disease epidemics and growth in the prevalence of chronic diseases – with emphasis on diabetes and hypertension, in addition to overweight and obesity. Identification of this transition has pointed to the existence of another transition, namely with regard to food, characterized by the replacement of home-produced meals with ready-to-eat or pre-prepared options (5).

Investigation of this new diet and its effects on health led to the creation of the Nova Food Classification. Developed in 2009 by the *Universidade de São Paulo* Center for Epidemiological Research in Nutrition and Health, the system organizes foods according to the degree and purpose of processing, creating four categories (fresh or minimally processed foods, culinary ingredients, processed foods and ultra-processed foods) (6-7). Since then, researchers around the world have been producing evidence that consumption of ultra-processed foods is associated with greater risk of developing a series of chronic diseases (8). 

From the academic world to the policy environment, the Nova Classification underwent a process to facilitate its comprehension, brought about by the second edition of the *Dietary Guidelines for the Brazilian Population* (9). This official Ministry of Health document adopted the Nova classification as the basis for its dietary guidelines. Its golden rule sums up its spirit: “Always prefer natural or minimally processed foods and culinary preparations over ultra-processed foods” (10). Following the publication, several public policies were implemented in Brazil and in countries inspired by Brazilian pioneering with the aim of reducing consumption of ultra-processed foods and expanding the population’s access to “real” food, encouraging adequate and healthy eating (11).

This narrative review, published a decade after the launch of the Guide, organizes information about its origin and historical context, analyzing its contributions to the science of Nutrition and public food and nutrition policies in Brazil and around the world. 

## Methods

In order to produce this narrative review, we consulted scientific articles in the PubMed and SciELO databases, as well as gray literature, such as technical documents available on Federal Government, state and municipal websites, and news articles in newspaper collections. Publications subsequent to the launch of the Guide were also collected, such as systematic reviews. The consultation covered publications from 2014 to July 2024, without language restrictions, encompassing national and international documents.

This review includes the critical and personal analysis of its authors, adding their views on the origin, trajectory and impacts of the Guide. This is justified by the close relationship between them and the Guide – notably in its design, implementation and scientific dissemination.

## Results

A first dimension of the results concerns the origin and historical context of the Guide. Whether due to the absence or excess of nutrients, food care has played a prominent role in solving global public health challenges for at least eight decades. Proof of this is the creation, in 1945, of the Food and Agriculture Organization of the United Nations, having as the primary objective of its mandate “raise levels of nutrition and people’s standard of living”. It was this same institution, together with the World Health Organization, that promoted the concept of food-based dietary guides in relation to public policies. This was announced at the International Conference on Nutrition in 1992, when strategies were discussed to improve nutritional well-being and food consumption worldwide. The event culminated in an action plan that emphasized the importance of countries developing their food guides individually, taking into account the public health challenges faced by the different Member States. It was an incentive for guides to start being organized around the world (12).

The purposes of the guides are clear: to promote autonomy in healthy food choices for individuals and communities, to be a health communication tool to convey dietary recommendations for the population, and to guide the actions of policy decision makers. By 2020, more than one hundred countries had published their food guides (13), with Brazil publishing its first edition of the document in 2006 (14) and the second in 2014 (9).

The design of a food guide is based on an understanding of the nature, extent, causes and possible solutions to public health problems related to food in a given territory. This understanding requires analysis of scientific evidence on the topic, consequent understanding of the state of the art and consideration of nutritional paradigms built and modified over time. An example of these paradigms is the definition of nutritional reference values ​​published in the 1950s (which established estimated daily amounts of energy and nutrients to meet the nutritional needs of individuals).

In Brazil, of course, the context in which the Guide was created involved some policy milestones related to food and nutrition that were being developed nationally. The trend was to be intensified during the two presidential terms of Luiz Inácio Lula da Silva – who made clear expressions of interest in combating hunger, even creating a ministry focused on the issue –, being continued by then president Dilma Rousseff in a subsequent term of office. The first of the milestones is the National Food and Nutrition Policy, published in 1999 (15). It attests to the commitment of the Brazilian National Health System (*Sistema Único de Saúde* - SUS) to food in two areas: hardship and harm related to food shortages and poverty, and excesses manifested through high prevalence of overweight and obesity which, at the time, already significantly affected the adult population (16).

The 1st edition of the Guide (14) contained theoretical concepts and seven guidelines related to nutrients and food groups, this being a very different approach from the 2nd edition of the document, as will be seen below. Two other important references emerged in 2010: the proposal of the Nova Food Classification, highlighting the role of food processing on its nutritional quality (17, 18) and Constitutional Amendment No. 64, recognizing food as a social right.

Between 2010 and 2011, the National Food and Nutritional Security Plan (19) emerged, in addition to the second edition of the National Food and Nutrition Policy (20), which reinforced its role within the SUS and as part of the intersectoral efforts that converge in the National Food and Nutrition Security System (16). The Policy has as one of its guidelines the Promotion of Adequate and Healthy Eating, understood as a set of strategies that provide individuals and communities with eating practices appropriate for their biological and sociocultural aspects, as well as the sustainable use of the environment. 

Promotion of Adequate and Healthy Eating should

“be in accordance with the needs of each stage of life and special dietary needs; be referenced by food culture and the dimensions of gender, race and ethnicity; be accessible from a physical and financial point of view; be balanced in quantity and quality; based on adequate and sustainable production practices; and with minimal amounts of physical, chemical and biological contaminants” (20).

This is an expanded vision that ended up determining the approach and principles for revising the Guide (21).

These factors happening together led to the publication of the 2nd edition of the Guide, in 2014, which is celebrated in this article. Despite being a significantly different document from the previous one, it started with the first edition, released in 2006, and was built by means of an intense review process. In this sense, it is worth highlighting the democratic nature of the preparation of the second edition, with listening and dialogue between different actors in society and analysis of suggestions collected through public consultation, which generated a report that was published together with the official Guide (22,23).

The Guide is innovative for several reasons, the main one being the incorporation of the Nova Food Classification as the basis of its dietary guidelines. For the first time, a food guide recognized the impacts of processing on food and diet quality. The second chapter of the document provides details on the four Nova categories, essential for understanding the golden rule of the Guide’s recommendations, which encourages a diet based on fresh or minimally processed foods and avoiding consumption of ultra-processed options. Furthermore, the Guide advocates variety, preference for foods of plant origin, encouragement of family farming, local economy and biodiversity and the importance of domestic cooking and food culture in the preparation and consumption of meals. The Guide also offers a contextual and realistic view of food, covering topics such as: food planning, ways of eating, household organization and food choices. Finally, it recognizes the challenges of maintaining an adequate and healthy diet, proposing solutions. The document was designed with Brazilian citizens as its target audience and, therefore, has attractive and easy-to-use textual and visual language (9,10).

Two years after the Guide was published, in 2016, the second edition of the National Food and Nutritional Security Plan (24) attested to the commitment of the Federal Government, the SUS and the National Food and Nutritional Security System to implementing its recommendations. This, however, was just the beginning of the Guide’s denouements, which had a series of positive impacts in Brazil and the world, raising on the global agenda discussion about the harmful potential of ultra-processed foods and the importance of a diet based on fresh foods. Next, we will analyze the repercussions of the Guide in two spheres: in the science of Nutrition and in public food and nutrition policies.

Regarding the Guide’s relationship with the science of Nutrition, it is important to highlight that it is a public policy instrument based on a set of scientific evidence (25). It encompasses an epistemological vision of how to understand the relationships between food, nutrition and health, gaining an item on the topic “Different sources of knowlege inform sound dietary advice” in a chapter entirely dedicated to its principles. As seen previously, the Guide was launched in 2014, five years after the first scientific publication on the role of food processing on its nutritional quality and health (17), which would quickly culminate in the Nova classification (18). By adopting the Nova classification so soon, the Guide gave rise to a set of studies on this food classification. Scientists from different countries began to dedicate themselves to producing knowledge on the subject, as demonstrated in the umbrella review by Lane et al. (8). 

Both Collaborative and global in nature, the aforementioned scientific work clarified several impacts of consumption of ultra-processed foods. One of them derives from the effect that ultra-processed foods have on replacing other foods in the diet, causing a double problem: their presence in the diet and their hindering consumption of fresh or minimally processed foods. This is because, once a person opts for ultra-processed meals, they will stop consuming other foods (26). This mechanism is related to the second impact, which indicates that ultra-processed foods compromise the nutritional quality of the diet, generating inadequate levels of free sugars, saturated fat and fiber (27). It was also found that ultra-processed foods induce excessive energy consumption for several reasons – including their low satiety power (28).

On a broader note, it is clear that dietary patterns rich in ultra-processed foods increase risk of disease and death in populations. In this sense, scientific research initially focused on cardiovascular diseases, diabetes and hypertension – these being conditions most clearly associated with diet. Years later, however, scientific literature was to point to links between ultra-processed foods and at least 32 human health problems (8), with some outcomes apparently being more distant from traditional metabolic pathways, such as different types of cancer (29) and depression (30). Finally, the Guide also took into account the impacts of ultra-processed dietary patterns on nature and food systems (31, 32). They can be summarized into three impacts: food monotony from farm to table, the increase in carbon, water and ecological footprints of food and the loss of diversity of animal and plant species used in food and agriculture. This last point incorporated into the Guide a holistic and systemic perspective of the mankind/food binomial.

**Figure 1:**
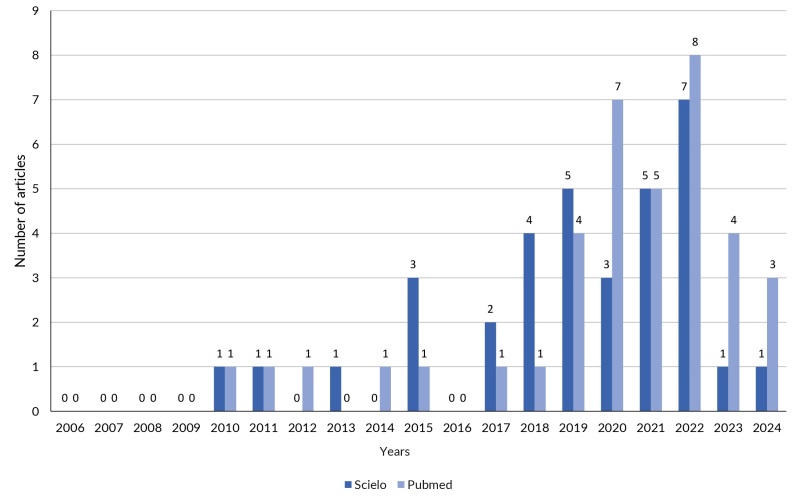
Articles published about the Guide, per database, 2006-2024

It is interesting to note that, naturally, scientific evidence on ultra-processed foods continued to be produced after the launch of the Guide. It appears that, ten years later, the vast and diverse scientific literature on the topic not only reinforces the recommendations contained in the Guide, but also brings new points of view, such as the already mentioned relationship between ultra-processed foods and mental health (30). The validity, quality and pioneering nature of the Guide’s content gave it the status of an object of scientific research. As shown in Figure 1, there has been an increase in scientific publications about the Guide, taking articles that used the terms “*Dietary Guidelines for the Brazilian Population*” or “*Brazilian Dietary Guidelines*” in their titles or abstracts. An example is the review conducted by researchers from the *Universidade Federal de Viçosa* that identifies and discusses tools for promoting and evaluating adequate and healthy eating based on the Guide (33). 

The implementation of the Guide formed part of the framework of public policies to promote healthy eating and, at the same time, its existence has been the basis for the development of new policies (34). Examples of this are: the 2nd National Food and Nutritional Security Plan (24), challenge 5 of which brings goals related to the implementation of the Guide’s recommendations, or the Strategic Action Plan for Addressing Chronic Diseases and Non-Communicable Diseases in Brazil 2021-2030, with its goal of reducing consumption of ultra-processed foods (35). 

Basically, implementation of food guides can be divided into two stages. The first is prior to their release, involving the preparation of its recommendations (which must be viable, understandable and culturally referenced) and simultaneous development of an integrated implementation plan. The second stage begins shortly after the guides are released, and considers guides from two aspects: as an educational material and as a promoter of public policies. In the case of the Brazilian Guide, it was used as a health education instrument through strategies such as the distribution of printed copies and availability of its digital version, production of materials and courses, and as support in training and dissemination processes (11). 

An example of this stage is the publication of the collection entitled “Protocols for using the Food Guide for the Brazilian Population in individual dietary guidance”, developed by researchers from the *Universidade de São Paulo* Center for Epidemiological Research in Nutrition and Health. They cover five issues aimed at audiences at different stages of the life cycle and with protocols that facilitate the application of the Guide’s principles in individual consultations carried out by various health professionals (not just nutritionists) (36-38). The collection also generated a qualification course in distance education format, QualiGuia - Training for the use of Protocols for the Use of the *Food Guide for the Brazilian Population*, developed within the scope of the SUS Institutional Development Support Program, being widely available on the internet course platform of the SUS Open University System. Furthermore, the Ministry of Health has developed, in partnership with public universities, various educational materials, such as manuals, instructions, folders and videos (11, 34). Among a set of publications, the revision of the *Dietary guidelines for Brazilian children under two years of age* is also mentioned, with guidelines aligned to the *Dietary Guidelines for the Brazilian Population* (44).

Regarding the Guide as a promoter of public policies, it has been essential for the formulation of federal and municipal measures. The first action occurred in 2015, when the Ministry of Health and related entities prohibited the offering, sale and advertising of ultra-processed foods in the workplace (40). In 2020, a National Education Development Fund resolution limited the purchase of ultra-processed products and encouraged consumption of fresh foods within the scope of the National School Meal Program (41). A series of policies were implemented in 2023, such as the Brazil Without Hunger Plan (advising that social protection actions for families in situations of food insecurity should be based on food guides) (42), the municipal laws of Rio de Janeiro (43) and Niterói (44), which prohibit the offer and sale of ultra-processed foods in public and private schools, and the publication of three presidential decrees. These regulate the new composition of the basic food basket (according to the Guide and the Nova classification) (45), guide actions to promote adequate and healthy eating in the school environment (46), as well as establishing the National Food Supply Policy and Plan (47).

The Guide’s principles have also served as a basis for the development of food guides in other countries, especially in Latin America (Uruguay, Peru, Ecuador, Mexico and Chile) (48-52), as well as in other regions, such as Canada (53) and India (54). It is interesting to note how some countries relied on the Brazilian Guide and, after that, developed other initiatives that inspire Brazil, in a virtuous exchange. One example is Mexico, which incorporated the Nova classification into its guide and launched a package of public policies involving taxation of sugary drinks and restrictions on advertising of ultra-processed products (55). Brazil is currently undergoing debates on tax reform, with great pressure from organized civil society for ultra-processed foods to be surtaxed. Complementary Bill of Law - PLP No. 68/2024, submitted by the Federal Government to the National Congress, proposes zero tariffs for food items comprising the basic food basket and a selective tax for sugary drinks, denoting consistency with the Guide (56). 

**Figure 2:**
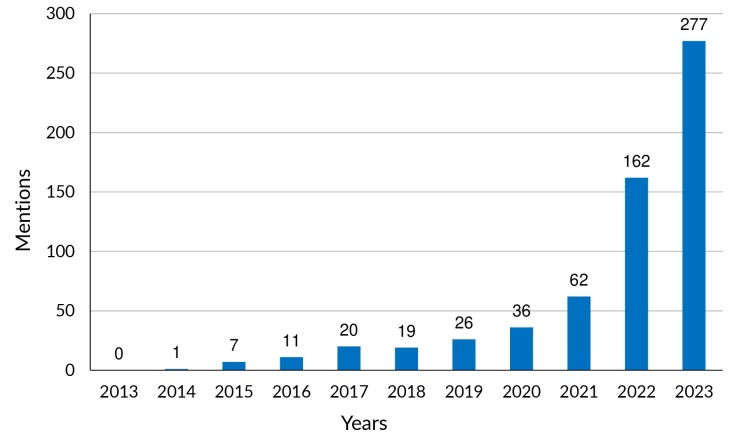
Mention of the term ultra-processed foods in the three largest printed Brazilian newspapers - O Estado de São Paulo, Folha de São Paulo and O Globo, 2013-2023

It is interesting to note that, at the same time that the Guide has influenced the formulation of public policies, use of the term ultra-processed foods in the Brazilian press has grown dramatically (Figure 2).

## Discussion

Ten years after the launch of the second edition of the Guide, its impact on both science and the formulation and implementation of public policies is clear. The adoption of the Nova food classification as the basis of its guidelines brought a paradigm shift in nutritional epidemiology that takes into consideration the harmful potential of ultra-processed foods for health and encourages a diet based on natural or minimally processed foods and culinary preparations. 

When analyzing its impact on science, it can be seen that the Guide consolidated evidence on the negative impacts of ultra-processed foods and became an object of research, serving as a basis for the creation of tools for promoting and evaluating adequate and healthy eating. Global scientific production has reinforced and expanded the Guide’s recommendations, demonstrating the relationship between consumption of ultra-processed foods and increased risk of developing various diseases. Over time, global scientific production has reinforced and expanded the Guide’s recommendations, demonstrating the relationship between consumption of ultra-processed foods and the increased risk of developing a variety of chronic non-communicable diseases.

Regarding public policies, the Guide has had great impact on promoting adequate and healthy eating, leading to the creation of a series of measures in Brazil and around the world. In Brazil, the implementation of the Guide has involved the development of health education strategies – initiatives that have facilitated the practical application of the Guide’s recommendations among the population, expanding its reach. The Guide has had repercussions on federal and municipal initiatives, such as the ban on the sale and advertising of ultra-processed foods in work environments, the limitation on the purchase of ultra-processed foods by the National School Food Program and municipal laws that prohibit the sale and supply of ultra-processed foods in schools. The international influence of the Brazilian Guide also stands out as an inspiration for the creation, in several countries, of guides that have adopted its principles and approaches in producing dietary recommendations for their populations.

However, there are challenges in disseminating the Guide. Examples are the lack of knowledge about it and consequent underuse of its guidelines by health professionals in primary care and in other parts of the health care network – such as hospital care. Insufficient incorporation of important topics, such as food sustainability, also persists.

The Guide’s trajectory shows the importance of a participatory and democratic process for preparing a document guiding adequate and healthy eating, involving different sectors of society and taking local food cultures into consideration, in addition to the relevance of the process of implementing the Guide, fundamental for its effectiveness. The broad and contextual perspective of nutrition proposed by the Guide has been able to induce and guide health actions and other public policies to promote adequate and healthy eating and has boosted scientific research, having national and global influence. Expanding the implementation of its guidelines is essential for addressing current and future public health and environmental challenges. 
