# Supplementary material for: Ten years of the The Dietary Guidelines for the Brazilian Population: history, science and policy
Source: Epidemiol Serv Saude. 2025 Mar 31;34:e20240267. doi: 10.1590/S2237-96222025v34e20240267.en (PMC11970360; doi:10.1590/S2237-96222025v34e20240267.en)
Supplement: Translation

# Translation: Dez anos do Guia Alimentar para a População Brasileira: história, ciência e política

**DOI:** 10.1590/S2237-96222025v34e20240267.pt

**Authors:** Patricia Constante Jaime, Murilo Bomfim Lobo Braga

**Affiliations:** 2 Universidade de São Paulo, Núcleo de Pesquisas Epidemiológicas em Nutrição em Saúde, São Paulo, SP, Brasil

**Keywords:** Guias Alimentares, Política Nutricional, Promoção da Saúde, Brasil, Revisão Narrativa

## Abstract

**Objetivo:**

Este artigo organiza a origem e o contexto histórico da segunda edição do *Guia Alimentar para a População Brasileira* dez anos depois de sua publicação, analisando suas contribuições para a ciência da Nutrição e para as políticas públicas de alimentação e nutrição no Brasil e no mundo.

**Métodos:**

Trata-se de uma revisão narrativa elaborada a partir da consulta de artigos científicos, literatura cinzenta e publicações jornalísticas, entre outras.

**Resultados:**

O Guia surgiu a partir de evidências científicas e, posteriormente, tornou-se objeto de estudo. Seu embasamento na Classificação Nova de Alimentos trouxe um novo paradigma para a ciência nutricional. Sua publicação foi base de uma série de políticas públicas que incentivam o consumo de alimentos in natura ou minimamente processados e mitigam o consumo de opções ultraprocessadas.

**Conclusão:**

O Guia foi capaz de induzir e orientar ações de saúde e de outras políticas públicas para promoção da alimentação adequada e saudável. Também impulsionou pesquisas científicas, tendo influência nacional e global.

## Introdução

Ao longo dos últimos 15 anos, vimos, no Brasil e em outros países (sobretudo na América Latina), uma mudança no paradigma da alimentação e da nutrição. Por décadas, o ensino, a pesquisa e as políticas de nutrição eram baseadas em uma linha de pensamento centrada em nutrientes: os alimentos eram classificados por seu conteúdo de macro e micronutrientes, e as orientações nutricionais visavam garantir a ingestão de quantidades mínimas de carboidratos, proteínas e gorduras, o que fazia sentido na maior parte do século XX (1-4).

A transição epidemiológica registrada no período mostrava a superação das epidemias de doenças infecciosas e o crescimento das prevalências de doenças crônicas — com destaque para diabetes e hipertensão, além dos quadros de sobrepeso e obesidade. A identificação dessa transição apontou a existência de uma outra: a alimentar, caracterizada pela substituição das refeições produzidas em casa por opções prontas ou pré-prontas (5).

A investigação dessa nova dieta e de seus efeitos na saúde levou à criação da Classificação Nova de Alimentos. Elaborado em 2009 pelo Núcleo de Pesquisas Epidemiológicas em Nutrição e Saúde da Universidade de São Paulo, o sistema organiza os alimentos segundo o grau e finalidade de processamento, criando quatro categorias (Alimentos in natura ou Minimamente Processados, Ingredientes Culinários, Alimentos Processados e Alimentos Ultraprocessados) (6-7). Desde então, pesquisadores em todo o mundo vêm produzindo evidências de que o consumo de alimentos ultraprocessados está associado a um maior risco de desenvolvimento de uma série de doenças crônicas (8). 

Da academia para o ambiente político, a Classificação Nova sofreu um processo de translação facilitado pela segunda edição do *Guia Alimentar para a População Brasileira* (9). Esse documento oficial do Ministério da Saúde adotou a classificação como base para suas diretrizes alimentares. Sua regra de ouro resume seu espírito: “Prefira sempre alimentos in natura ou minimamente processados e preparações culinárias a alimentos ultraprocessados” (10). A partir da publicação, diversas políticas públicas foram implementadas no Brasil e em países inspirados pelo pioneirismo brasileiro com o objetivo de reduzir o consumo de ultraprocessados e ampliar o acesso das populações à comida “de verdade”, incentivando uma alimentação adequada e saudável (11).

Esta revisão narrativa, publicada uma década após o lançamento do Guia, organiza a origem e o contexto histórico do documento, analisando suas contribuições para a ciência da Nutrição e para as políticas públicas de alimentação e nutrição no Brasil e no mundo. 

## Métodos

Para a revisão narrativa, foram consultados artigos científicos das bases de dados PubMed e SciELO, além de literatura cinza, como documentos técnicos em sites do Governo Federal, de estados e municípios, e notícias nos acervos de jornais. Foram, ainda, levantadas publicações posteriores ao lançamento do Guia, como revisões sistemáticas. A consulta considerou publicações a partir de 2014 até julho de 2024, sem restrição de idioma, englobando documentos nacionais e internacionais.

Esta revisão inclui a análise crítica e pessoal de seus autores, adicionando suas visões sobre a origem, a trajetória e os impactos do Guia. Isso se justifica pela íntima relação de cada um deles com o Guia — notadamente na concepção e implementação e na divulgação científica do mesmo. 

## Resultados

Uma primeira dimensão dos resultados diz respeito à origem e contexto histórico do Guia. Seja pela ausência ou pelo excesso de nutrientes, o cuidado com a alimentação vem ocupando papel destaque na solução de desafios da saúde pública global há pelo menos oito décadas. Prova disso é a criação, em 1945, da Organização das Nações Unidas para Alimentação e Agricultura, que tem como primeiro objetivo de seu mandato o “aumento do nível de nutrição e de vida das pessoas”. Foi esta mesma instituição, junto à Organização Mundial da Saúde, que promoveu o conceito de guias alimentares baseados em alimentos no espaço das políticas públicas. A divulgação se deu na Conferência Internacional de Nutrição, em 1992, quando se discutiam estratégias para melhorar o bem-estar nutricional e o consumo de alimentos no mundo. O evento culminou em um plano de ação que enfatizava a importância de países desenvolverem seus guias alimentares individualmente, levando em conta os desafios de saúde pública enfrentados pelos distintos Estados. Era um incentivo para que os guias começassem a ser organizados mundo afora (12).

As funções dos guias são claras: promover a autonomia nas escolhas alimentares saudáveis para indivíduos e comunidades, ser ferramenta de comunicação em saúde para traduzir recomendações alimentares para a população, e orientar as ações dos tomadores de decisões políticos. Até 2020, mais de cem países publicaram seus guias alimentares (13), tendo o Brasil publicado sua primeira edição do documento em 2006 (14) e a segunda em 2014 (9).

A concepção de um guia alimentar se baseia na compreensão sobre a natureza, a extensão, as causas e as possíveis soluções para os problemas de saúde pública relacionados à alimentação em um determinado território. Essa compreensão exige a análise de evidências científicas sobre o tema, o consequente entendimento do estado da arte e a consideração de paradigmas nutricionais construídos e modificados ao longo do tempo. Um exemplo desses paradigmas é a definição de valores nutricionais de referência publicadas na década de 1950 (que estabeleceram quantidades diárias estimadas de energia e nutrientes para a satisfação de necessidades nutricionais de indivíduos).

No Brasil, é claro, o contexto de construção do Guia envolveu alguns marcos da política relacionada à alimentação e nutrição que vinha sendo desenvolvida no país. A tendência seria intensificada nos dois mandatos presidenciais de Luiz Inácio Lula da Silva — que fazia manifestações claras de interesse no combate à fome, criando, inclusive, um ministério voltado ao tema —, sendo continuada pela então presidente Dilma Rousseff em mandato subsequente. O primeiro dos marcos é a Política Nacional de Alimentação e Nutrição, publicada em 1999 (15). Ela atesta o compromisso do Sistema Único de Saúde (SUS) com a alimentação em duas linhas: os males relacionados à escassez alimentar e à pobreza e os excessos configurados pelas altas taxas de prevalência de sobrepeso e obesidade que, na época, já atingiam significativamente a população adulta (16).

A 1ª edição do Guia (14) trazia conceitos teóricos e sete diretrizes relacionadas a nutrientes e grupos alimentares — uma abordagem bastante distinta da 2ª edição do documento, como se verá adiante. Duas outras referências importantes surgiram em 2010: a proposição da Classificação Nova de Alimentos, destacando o papel do processamento de alimentos em sua qualidade nutricional (17,18) e a Emenda Constitucional nº 64, reconhecendo a alimentação como um direito social.

Entre 2010 e 2011, surgiu o Plano Nacional de Segurança Alimentar e Nutricional (19), além da segunda edição da Política Nacional de Alimentação e Nutrição (20), que reforçou o seu papel junto ao SUS e como integrante dos esforços intersetoriais que confluem no Sistema Nacional de Segurança Alimentar e Nutricional (16). A Política tem como uma de suas diretrizes a Promoção da Alimentação Adequada e Saudável, entendida como um conjunto de estratégias que proporciona, aos indivíduos e coletividades, a realização de práticas alimentares apropriadas aos seus aspectos biológicos e socioculturais, bem como ao uso sustentável do meio ambiente. 

A Promoção da Alimentação Adequada e Saudável deve

“estar em acordo com as necessidades de cada fase do curso da vida e com as necessidades alimentares especiais; ser referenciada pela cultura alimentar e pelas dimensões de gênero, raça e etnia; ser acessível do ponto de vista físico e financeiro; ser harmônica em quantidade e qualidade; baseada em práticas produtivas adequadas e sustentáveis; e com quantidades mínimas de contaminantes físicos, químicos e biológicos” (20).

Trata-se de uma visão ampliada que acabou por determinar a abordagem e os princípios para a revisão do Guia (21).

A concomitância desses fatores levou à publicação da 2ª edição do Guia, em 2014, celebrada no presente artigo. Apesar de ser um documento significativamente distinto do anterior, ele principia na primeira edição, de 2006, sendo construído em um intenso processo de revisão. Nesse sentido, vale destacar o caráter democrático da elaboração da segunda edição, com escutas e diálogos entre diversos atores da sociedade e a análise de sugestões coletadas em consulta pública, o que gerou um relatório publicado junto ao documento oficial (22,23).

O Guia é inovador por diversas razões — a principal delas é a incorporação da Classificação Nova de Alimentos como base de suas orientações alimentares. Pela primeira vez, um guia alimentar reconhecia os impactos do processamento nos alimentos e na qualidade da dieta. O segundo capítulo do documento traz detalhamentos sobre as quatro categorias da Nova, essenciais para o entendimento da regra de ouro das recomendações, que incentiva a alimentação baseada em alimentos in natura ou minimamente processados e que se evite o consumo de opções ultraprocessadas. Além disso, o Guia preconiza a variedade, a preferência por alimentos de origem vegetal, o estímulo à agricultura familiar, à economia local e à biodiversidade e a importância da culinária doméstica e da cultura alimentar no preparo e consumo de refeições. O documento oferece, ainda, uma visão contextual e realista da alimentação, abarcando temas como: o planejamento alimentar, os modos de comer, a organização doméstica e as escolhas dos alimentos. Por fim, reconhece os desafios da manutenção da alimentação adequada e saudável, propondo soluções. O documento foi pensado tendo o cidadão brasileiro como público-alvo e, por isso, tem linguagem textual e visual atraente e facilitada (9, 10).

Dois anos após a publicação do Guia, em 2016, a segunda edição do Plano Nacional de Segurança Alimentar e Nutricional (24) atestou o compromisso do Governo Federal, do SUS e do Sistema Nacional de Segurança Alimentar e Nutricional com a implementação de suas recomendações. Este, no entanto, foi apenas o início dos desdobramentos do Guia, que teve uma série de impactos positivos no Brasil e no mundo, inserindo a discussão sobre o potencial nocivo dos alimentos ultraprocessados e a importância da alimentação baseada em alimentos in natura na agenda global. A seguir, analisaremos as repercussões do Guia em duas esferas: na ciência da Nutrição e nas políticas públicas de alimentação e nutrição.

No tocante à relação do Guia com a ciência da Nutrição, é importante destacar que ele é um instrumento de política pública baseado em um conjunto de evidências científicas (25). Ele abarca uma visão epistemológica de como compreender as relações entre alimentação, nutrição e saúde, ganhando um item sobre o tema “Diferentes saberes geram o conhecimento para a formulação de guias alimentares” em capítulo inteiramente dedicado aos seus princípios. Como se viu anteriormente, o Guia foi lançado em 2014, cinco anos após a primeira publicação científica sobre o papel do processamento de alimentos em sua qualidade nutricional e na saúde (17), o que rapidamente culminaria na classificação Nova (18). Ao adotar tão rapidamente a Nova, o Guia impulsionou um conjunto de estudos sobre essa classificação de alimentos. Cientistas de diversos países passaram a se dedicar à produção de conhecimento a respeito do assunto, como demonstrado na revisão guarda-chuva de Lane e colaboradores (8). 

Colaborativo e global, esse fazer científico esclareceu diversos impactos do consumo de alimentos ultraprocessados. Um deles vem do efeito que os ultraprocessados têm de substituir outros alimentos na dieta, causando um duplo problema: sua presença na dieta e a inibição do consumo de alimentos in natura ou minimamente processados. Isso porque, uma vez que o comensal optou pela refeição ultraprocessada, deixará de consumir outros alimentos (26). É um mecanismo relacionado ao segundo impacto, que indica que alimentos ultraprocessados comprometem a qualidade nutricional da dieta, gerando uma inadequação de açúcares livres, gordura saturada e fibras (27). Também se constatou que os ultraprocessados induzem consumo excessivo de energia por diversos motivos — entre eles, seu baixo poder de saciedade (28).

Em uma observação mais ampla, fica claro que os padrões alimentares ricos em alimentos ultraprocessados aumentam o risco de doenças e de morte nas populações. Nesse sentido, inicialmente as pesquisas científicas focaram em doenças cardiovasculares, diabetes e hipertensão — patologias mais claramente associadas à alimentação. Anos depois, no entanto, a literatura científica viria a sinalizar nexos entre ultraprocessados e pelo menos 32 agravos à saúde humana (8), sendo alguns desfechos aparentemente mais distantes das tradicionais vias metabólicas, como os diversos tipos de câncer (29) e depressão (30). Por fim, o Guia também levou em conta os impactos dos padrões alimentares ricos em ultraprocessados na natureza e nos sistemas alimentares (31,32). Eles podem ser resumidos em três: a monotonia alimentar do campo à mesa, o aumento das pegadas de carbono, hídrica e ecológica da alimentação e a perda da diversidade de espécies animais e vegetais utilizadas na alimentação e na agricultura. Este último ponto incorporou ao Guia uma perspectiva holística e sistêmica do binômio homem/alimento.

É interessante notar que, naturalmente, as evidências científicas sobre ultraprocessados continuaram a ser produzidas após o lançamento do Guia. Constata-se que, dez anos depois, a vasta e diversa literatura científica sobre o tema não apenas reforça as recomendações contidas no documento, como traz novos pontos de vista, a exemplo da já citada relação entre ultraprocessados e saúde mental (30). A validade, a qualidade e o pioneirismo do conteúdo do Guia conferiram a ele o caráter de objeto de pesquisa científica. Como demostrado na Figura 1, houve aumento de publicações científicas sobre o Guia, considerando artigos que tiveram uso dos termos “*Dietary Guidelines for the Brazilian Population*” ou “*Brazilian Dietary Guidelines*” no título ou resumo. Um exemplo é a revisão conduzida por pesquisadoras da Universidade Federal de Viçosa que identifica e discute ferramentas para promoção e avaliação de alimentação adequada e saudável baseadas no documento (33). 

**Figura 1:** Artigos publicados sobre o Guia, segundo base de dados, de 2017-2024

A implementação do Guia integra o arcabouço de políticas públicas de promoção da alimentação saudável e, ao mesmo tempo, sua existência é base para o desenvolvimento de novas políticas (34). Exemplos disso são: o 2º Plano Nacional de Segurança Alimentar e Nutricional (24), que, em seu desafio 5, traz metas relacionadas à implementação das recomendações do Guia, ou o Plano de Ações Estratégicas para o Enfrentamento das Doenças Crônicas e Agravos Não Transmissíveis no Brasil 2021-2030, com meta de redução do consumo de ultraprocessados (35). 

Basicamente, a implementação de guias alimentares pode ser dividida em dois momentos. O primeiro é prévio ao lançamento, com a própria elaboração das recomendações (que devem ser viáveis, compreensíveis e culturalmente referenciadas) e o simultâneo desenvolvimento de um plano de implementação integrado. O segundo momento inicia logo após o lançamento, e considera o guia sob dois aspectos: como um material educativo e como um promotor de políticas públicas. No caso do guia brasileiro, o documento foi utilizado como instrumento de educação em saúde sob estratégias como a distribuição de cópias físicas e disponibilização da versão digital, a produção de materiais e cursos, e como apoio em processos de formação e disseminação (11). 

Um exemplo desta etapa é a publicação da coleção *Protocolos de uso do Guia Alimentar para a População Brasileira* na orientação alimentar individual, desenvolvido por pesquisadoras do Núcleo de Pesquisas Epidemiológicas em Nutrição e Saúde da Universidade de São Paulo. Com cinco fascículos voltados a públicos em diferentes estágios dos ciclos da vida e com protocolos que facilitam a aplicação dos preceitos do Guia em consultas individuais realizadas por diversos profissionais da saúde (não apenas nutricionistas) (36-38). A coleção também gerou um curso de qualificação no formato de educação à distância, o QualiGuia - Formação para utilização dos *Protocolos de Uso do Guia Alimentar para a População Brasileira*, desenvolvido no âmbito do Programa de Apoio ao Desenvolvimento Institucional do SUS, sendo amplamente disponibilizado na plataforma de cursos do Sistema Universidade Aberta do SUS. Além disso, o Ministério da Saúde desenvolveu, em parceria com universidades públicas, diversos materiais educativos, como manuais, instrutivos, folders e vídeos (11,34). Cita-se, ainda, em meio a um conjunto de publicações, a revisão do *Guia Alimentar para Crianças Brasileiras Menores de Dois Anos*, com diretrizes alinhadas ao Guia (44).

No que tange ao Guia como promotor de políticas públicas, o documento foi essencial para a elaboração de medidas federais e municipais. A primeira ação ocorreu em 2015, quando o Ministério da Saúde e entidades vinculadas proibiram a oferta, venda e publicidade de alimentos ultraprocessados em ambiente de trabalho (40). Em 2020, uma resolução do Fundo Nacional de Desenvolvimento da Educação limitou a compra de produtos ultraprocessados e incentivou o consumo de alimentos in natura no âmbito do Programa Nacional de Alimentação Escolar (41). Uma série de políticas foram implementadas em 2023, como o Plano Brasil sem Fome (orientando que ações de proteção social para famílias em situação de insegurança alimentar devem se basear nos guias alimentares) (42), as leis municipais do Rio de Janeiro (43) e de Niterói (44) que proíbem a oferta e a venda de ultraprocessados em escolas das redes pública e privada, e a publicação de três decretos presidenciais. Estes regulamentam a nova composição da cesta básica (de acordo com o Guia e a classificação Nova) (45), orientam as ações de promoção da alimentação adequada e saudável no ambiente escolar (46) e instituem a Política e o Plano Nacional de Abastecimento Alimentar (47).

Os preceitos do Guia também serviram de base para a elaboração de guias alimentares de outros países, sobretudo na América Latina (Uruguai, Peru, Equador, México e Chile) (48-52), mas também de outras regiões, a exemplo do Canadá (53) e da Índia (54). É interessante notar como alguns países se apoiaram no Guia brasileiro e, depois disso, desenvolveram outras iniciativas que inspiram o Brasil, em um intercâmbio virtuoso. Um exemplo é o México, que incorporou a classificação Nova em seu guia e lançou um pacote de políticas públicas envolvendo taxação de bebidas açucaradas e restrições à publicidade de ultraprocessados (55). Neste momento, o Brasil passa por debates sobre a reforma tributária, com grande pressão da sociedade civil organizada para que alimentos ultraprocessados sejam sobretaxados. O Projeto de Lei Complementar - PLP nº68/2024, apresentado pelo Governo Federal ao Congresso Nacional, propõe tarifa zero para os alimentos da cesta básica e imposto seletivo para bebidas açucaradas, denotando coerência com o Guia (56). 

É interessante notar que, ao mesmo tempo em que o Guia influenciava a construção de políticas públicas, aparições do termo alimentos ultraprocessados na imprensa brasileira cresciam vertiginosamente (Figura 2).

**Figura 2:** Menções do termo alimentos ultraprocessados nos três jornais brasileiros impressos de maior circulação - O Estado de São Paulo, Folha de São Paulo e O Globo, de 2013-2023

## Discussão

Dez anos depois do lançamento da segunda edição do Guia, é nítido o impacto do documento tanto na ciência quanto na formulação e implementação de políticas públicas. A adoção da classificação de alimentos Nova como base de suas diretrizes trouxe uma mudança de paradigma na epidemiologia nutricional que considera o potencial nocivo à saúde dos alimentos ultraprocessados e incentiva uma alimentação baseada em alimentos in natura ou minimamente processados e em preparações culinárias. 

Quando se analisa o seu impacto na ciência, vê-se que o documento consolidou evidências sobre os impactos negativos dos ultraprocessados e se tornou objeto de pesquisa, servindo de base para a criação de ferramentas para promoção e avaliação da alimentação adequada e saudável. A produção científica global reforçou e expandiu as recomendações do Guia, demonstrando as relações entre o consumo de ultraprocessados e o aumento do risco de desenvolvimento de diversas doenças. Ao longo do tempo, a produção científica global reforçou e expandiu as recomendações do Guia, demonstrando as relações entre o consumo de ultraprocessados e o aumento do risco de desenvolvimento de diversas doenças crônicas não transmissíveis.

No que tange às políticas públicas, o Guia teve grande impacto na promoção de uma alimentação adequada e saudável, levando à criação de uma série de medidas no Brasil e no mundo. No Brasil, a implementação do Guia envolveu a elaboração de estratégias de educação em saúde — iniciativas que facilitaram a aplicação prática das recomendações do Guia junto à população, ampliando seu alcance. O documento teve reflexos em iniciativas federais e municipais, a exemplo da proibição de venda e publicidade de ultraprocessados em ambientes de trabalho, da limitação da compra de ultraprocessados pelo Programa Nacional de Alimentação Escolar e das leis municipais que proíbem a venda e a oferta de ultraprocessados nas escolas. Destaca-se, ainda, a influência internacional do Guia brasileiro como inspiração para a criação, em diversos países, de guias que adotaram seus princípios e abordagens na produção de recomendações alimentares para populações.

Notam-se, no entanto, desafios na disseminação do documento. Exemplos são o desconhecimento e a consequente subutilização de suas diretrizes por profissionais de saúde da atenção primária e em outros pontos da rede de atenção à saúde – como a atenção hospitalar. Também persiste a insuficiente incorporação de temas importantes, como a sustentabilidade na alimentação.

A trajetória do Guia mostra a importância de um processo participativo e democrático para a elaboração de um documento orientador da alimentação adequada e saudável, envolvendo diversos setores da sociedade e considerando as culturas alimentares locais, além da relevância do processo de implementação do documento, fundamental para sua efetividade. A perspectiva ampla e contextual da alimentação proposta pelo Guia foi capaz de induzir e orientar ações de saúde e de outras políticas públicas para promoção da alimentação adequada e saudável e impulsionou pesquisas científicas, tendo influência nacional e global. A expansão da implementação de suas diretrizes é essencial para o enfrentamento dos desafios atuais e futuros da saúde pública e do meio ambiente.
